# Identification of Service Areas for Nurseries Serving Mildly Ill Children in 105 Major Japanese Cities before and during the COVID-19 Pandemic

**DOI:** 10.31662/jmaj.2025-0555

**Published:** 2026-06-19

**Authors:** Akira Ehara

**Affiliations:** 1 Department of Health Services Management, Faculty of Health and Wellness Sciences, Hiroshima International University, Higashihiroshima, Hiroshima, Japan

**Keywords:** COVID-19, infection, Japan, nursery, service area

## Abstract

**Introduction:**

To prevent the spread of infectious diseases, general day care centers in Japan ask children to not attend when they are feeling unwell, even if they only have mild symptoms. Thus, when a child becomes ill, their continuity of childcare is disrupted. For this reason, specialized nurseries for children who are mildly ill have been established in Japan, aiming to ensure continuity of care. However, there is limited knowledge regarding the impact on nursery use caused by the distance between a child’s residence and the nearest facility.

**Methods:**

This survey targeted 105 major cities nationwide: 20 designated cities, 23 special wards of Tokyo, and 62 core cities. Using panel data analysis with generalized estimating equations, I determined the monthly number of users of day care for mildly ill children from April 2018 to March 2023 based on the average shortest distance in each city from each child’s residence to the corresponding facility. The analysis was adjusted for factors that included general infectious disease trends, childcare capacity for mildly ill children, child population, the coronavirus disease 2019 (COVID-19) pandemic, and presence/absence of a declaration of state emergency/priority infection control measures.

**Results:**

Both before and during the COVID-19 pandemic, the utilization rate decreased by a factor of 0.825 for every 1-km increase (approximately 17.5% decrease per kilometer) in the shortest distance to a nursery for mildly ill children. When the distance reached 12 km, the utilization rate decreased to 10%.

**Conclusions:**

Defining the service area as the distance range where utilization rates become one-tenth to equivalent compared with childcare facilities in the same neighborhood as a child’s home, the radius remained approximately 12 km both before and during the COVID-19 pandemic.

## Introduction

Infants and young children are susceptible to infectious diseases, and it is estimated that in Japan, this population visits a medical institution at least once a month for these illnesses ^(1)^. To prevent the spread of infectious diseases (listed in Table 1) ^(2)^, general day care centers in the country ask children not to attend when they are feeling unwell, even if they only have mild symptoms. Consequently, when a child becomes ill, their continuity of childcare is disrupted. For this reason, specialized nurseries for mildly ill children have been established in Japan, aiming to ensure continuity of care.

**Table 1.** Diseases Mentioned in the Guidelines for Prevention of Infectious Diseases in Day Care Centers and Handling Under the Infectious Disease Control Law.

Diseases mentioned in the Guidelines	Handling under the Infectious Disease Control Law	
	Category	Notification
Requires a written opinion from a physician		
*Influenza (excluding avian influenza and novel influenza or re-emerging influenza; x_4it_^#^)	V	^$^Influenza sentinel sites
*Pharyngoconjunctival fever (x_6it_)	V	Pediatric sentinel sites
*Varicella (x_9it_)	V	Pediatric sentinel sites
*Mumps (x_14it_)	V	Pediatric sentinel sites
*Epidemic keratoconjunctivitis (x_15it_)	V	Ophthalmology sentinel sites
*Acute hemorrhagic conjunctivitis (x_16it_)	V	Ophthalmology sentinel sites
Measles	V	Full
COVID-19	II (until May 7, 2023); V (on or after May 8, 2023)	Full (until May 7, 2023); ^$^influenza/COVID-19 sentinel sites (on or after May 8, 2023)
Rubella	V	Full
Tuberculosis	II	Full
Pertussis	V	Full
Enterohemorrhagic *Escherichia coli* infection	III	Full
Invasive meningococcal infection	V	Full
Requires a doctor’s note and parent/guardian’s notification of attendance		
*Respiratory syncytial virus infection (x_5it_)	V	Pediatric sentinel sites
*Group A streptococcal pharyngitis (x_7it_)	V	Pediatric sentinel sites
*Infectious gastroenteritis (x_8it_)	V	Pediatric sentinel sites
*Hand, foot, and mouth disease (x_10it_)	V	Pediatric sentinel sites
*Erythema infectiosum (x_11it_)	V	Pediatric sentinel sites
*Exanthema subitem (x_12it_)	V	Pediatric sentinel sites
*Herpangina (x_13it_)	V	Pediatric sentinel sites
Mycoplasma pneumonia	V	Designated sentinel sites
Herpes zoster	NA	NA

NA: not applicable *This analysis used the incidence rates of these infectious diseases. ^#^The notations from “x_4it_” to “x_16it_” are based on the description in the Materials and Methods section. ^$^The influenza sentinel sites were changed to the influenza/COVID-19 sentinel sites on May 8, 2023, and subsequently to the acute respiratory infection sentinel sites on April 7, 2025.

Users of the aforementioned childcare services have been reported to exhibit symptoms such as fever, cough, diarrhea, and vomiting ^(3)^. The number of users of these nurseries reportedly declined sharply during the coronavirus disease 2019 (COVID-19) pandemic ^(4)^. However, knowledge is limited regarding the impact on nursery use caused by the distance between a child’s residence and the nearest specialized nursery for mildly ill children.

All 105 major cities in Japan (20 designated cities, 23 special wards of Tokyo, and 62 core cities) provide financial assistance for day care for mildly ill children, maintain their own public health centers to monitor infectious disease trends, and manage records related to these operations. Therefore, using these records and adjusting for factors such as general infectious disease trends, childcare capacity for mildly ill children, and infant and toddler populations, it is possible to conduct a quantitative analysis of the relationship between childcare service use and the distance between a child’s home and a nursery specialized in caring for mildly ill children. Furthermore, although the utilization rate of public transportation such as taxis differs between urban and rural areas ^(5)^, these major cities are located in densely populated areas, so only slight differences in the modes of transportation used are expected. This makes these cities suitable for analyzing the impact of distance on childcare utilization.

I conducted an analysis covering 105 major cities nationwide to quantitatively clarify the impact of the distance from the child’s residence to a nursery for mildly ill children on the utilization rate of these facilities.

## Materials and Methods

### Target cities and analysis period

This study targeted 105 major cities nationwide: 20 designated cities, 23 special wards of Tokyo, and 62 core cities.

The analysis period covered each month from April 2018 to March 2023. The boundary between the prepandemic and pandemic periods for COVID-19 was defined as February 28, 2020, when the Ministry of Education, Culture, Sports, Science and Technology ordered the simultaneous closure of elementary, junior high, and high schools, as well as special needs schools. According to previous research ^(6)^, the use of nurseries for mildly ill children in 20 designated cities has declined sharply since March 2020. Information regarding the presence or absence of a declaration of state emergency/priority infection control measures was obtained from the cabinet office website ^(7)^.

### Questionnaire survey on the capacity and number of monthly users of nurseries for mildly ill children in 105 major cities

This study targeted nurseries that both provide day care for children with mild illnesses and receive municipal subsidies. I did not include childcare centers that serve healthy children but that continue to provide care for a child who becomes ill until the child’s regular departure time because these centers provide special care for mildly ill children only on the day symptoms appear. Furthermore, childcare services for mildly ill children outside of facilities (childcare services dispatching childcare workers to the child’s home) were also excluded. This is because the list provided by the Children and Families Agency from fiscal years 2019 to 2021 contained only six facilities nationwide.

Data on the maximum capacity of day care service facilities for mildly ill children as of April each year from 2018 to 2022, along with the monthly number of users of these services from April 2018 to March 2023, were collected via survey forms. These forms were mailed to all designated cities and special wards of Tokyo on June 26, 2023, and to core cities on December 5, 2024. For municipalities that did not respond, questionnaires were sent again 1 month and 2 months after the initial mailing as a reminder, with collection concluded after 3 months.

### Infectious disease surveillance data

Public health centers have been established in each of the 105 major cities of Japan, and these centers monitor and collect data on infectious disease incidence at numerous sentinel sites. This includes data for 13 infectious diseases commonly seen in children: influenza; respiratory syncytial virus infection; pharyngoconjunctival fever; group A streptococcal infection; infectious gastroenteritis; varicella; hand, foot, and mouth disease; erythema infectiosum; exanthema subitum; herpangina; mumps; epidemic keratoconjunctivitis; and acute hemorrhagic conjunctivitis (Table 1).

I requested epidemiological survey data on the aforementioned 13 infectious diseases, compiled from 105 municipal public health centers for the Ministry of Health, Labour and Welfare. The incidence rate of disease is reported on a weekly basis, which was converted to a monthly rate. When a week spanned 2 months, the rate was prorated.

### Calculating the distance from the residence to the nearest nursery for mildly ill children

**Figure 1.**
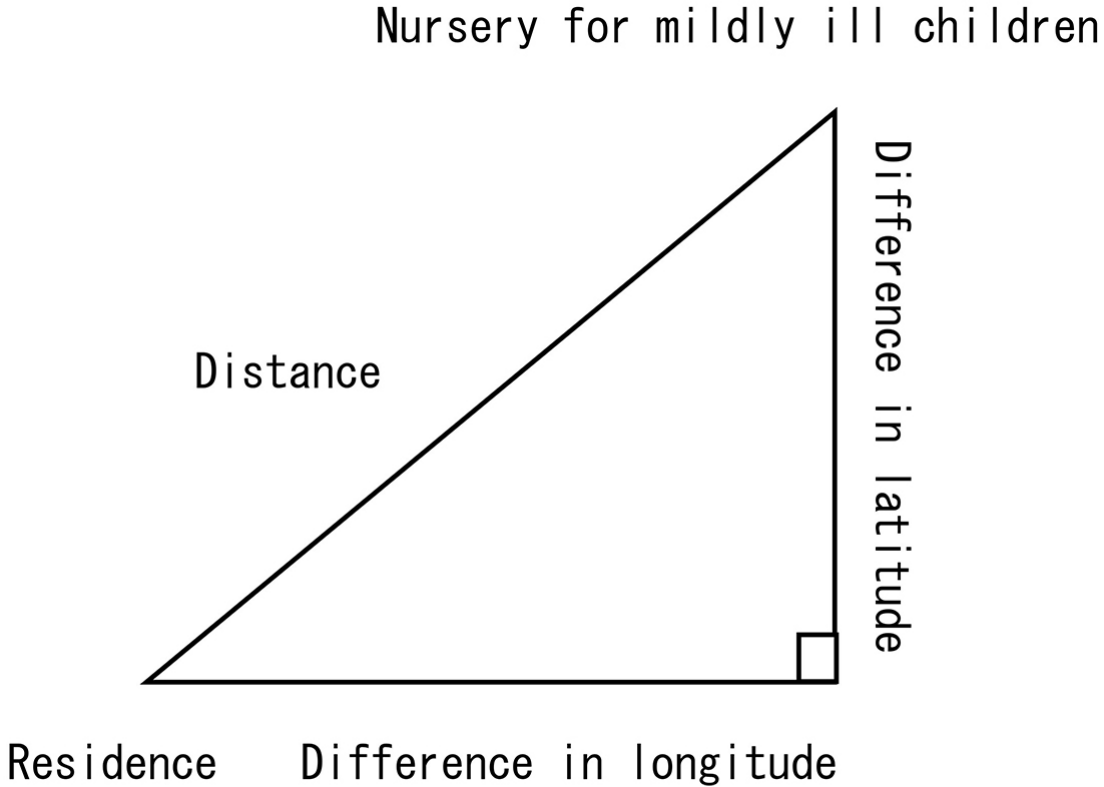
Calculation of distance from the residence to the nearest nursery for mildly ill children, using the difference in latitude and longitude and the Pythagorean theorem.

The shortest distance from a child’s residence to the nearest nursery for mildly ill children was calculated based on prior research using the difference in latitude and longitude and the Pythagorean theorem (Figure 1) ^(8)^.

I obtained lists of nurseries for mildly ill children from the Children and Families Agency. The facility list prior to the COVID-19 pandemic was designated as the fiscal year 2019 edition, and the list during the pandemic period was denoted the fiscal year 2021 edition. The locations of these nurseries were identified through each municipality’s website and converted to latitude and longitude using the geographic database provided by The University of Tokyo’s Center for Spatial Information Science ^(9)^.

The latitude and longitude for the residence of children aged 0 to 6 years are based on 500-meter mesh data created by dividing the entire country into 500-meter square grids ^(10)^. As of January 2025, the latest data for each 500-meter grid provided only 5-year forward population projections based on the 2015 census, with no annual population changes estimated ^(10)^. Therefore, calculations were performed using the 2020 estimates. The population aged 0 to 6 years within each mesh was used as a weighting coefficient to calculate the weighted average of the shortest distance in each city from a child’s residence to the nearest nursery for mildly ill children ^(8)^.

### Panel data analysis

Monthly fluctuations in the actual number of users of day care services for mildly ill children cannot be fully explained using a limited set of factors alone. Therefore, I estimated the number of users based on several factors and assumed that the actual user counts collected via the survey form were statistically distributed around these estimates. To determine the monthly number of users of these services in each city from April 2018 to March 2023, I conducted panel data analysis using generalized estimating equations under an exchangeable correlation structure based on the negative binomial distribution ^(11)^. Because the number of users is a positive integer, this analysis was performed based on the natural logarithm link function, using the following model:

log λ_it_ = a_0_ × x_0t_ + a_1_ × x_1it_ + (a_2_ + b_2_ × x_0t_) × x_2it_ + a_3_ × x_3it_ + (a_4_ + b_4_ × x_0t_) × x_4it_ + ... + (a_16_ + b_16_ × x_0t_) × x_16it_ + a_17_ × x_17i_ + c_ 0_ (constant)

where λ_it_ represents the predicted number of users (persons/month) of day care services for mildly ill children in their municipality i (i = 1-105) during month t (t = 1-60, April 2018 = 1, and March 2023 = 60); the actual number of users reported by each municipality was assumed to be the result of this estimate (λ_it_), fluctuating according to a negative binomial distribution. x_0t_ is the COVID-19 pandemic dummy variable (prepandemic = 0 and pandemic = 1); x_1it_ is the dummy variable for a declaration of state emergency/priority infection control measures (absent = 0 and present = 1); x_2it_ is the weighted average of the shortest distances (km) in each city from residential areas to nurseries for mildly ill children; and x_3it_ is the natural logarithm of the maximum capacity (persons/day) of day care service facilities for mildly ill children as of April each year from 2018 to 2022. The variables in x_4i_-x_16it_ represent the incidence rates (number of cases/sentinel site/month) of the 13 infectious diseases frequently observed in children (Table 1). x_17i_ is the natural logarithm of the population aged 0 to 6 years as of 2020 (persons, offset term); x_0t_ × x_2it_ is the product of the COVID-19 pandemic dummy variable and the weighted average of the shortest distances (km). The variables x_0t_ × x_4it_-x_0t_ × x_16it_ represent the product of the COVID-19 pandemic dummy variable and the incidence rates of the 13 infectious diseases (number of cases/sentinel site/month). The partial regression coefficients a_0_-a_17,_ b_2_, and b_4_-b_16_ are the coefficients of x_0t_-x_17i_, x_0t_ × x_2it_, and x_0t_ × x_4it_-x_0t_ × x_16it_, respectively.

Significance was determined with a p value <0.05.

### Ethics review

Although no personal information was handled in this study, consultation with the Ethics Committee for Life Science and Medical Research Involving Human Subjects, Hiroshima International University, regarding the need for review resulted in a determination that ethics review was not required (ethics review number 23-012, dated June 20, 2023, partially revised December 3, 2024).

## Results

**Figure 2.**
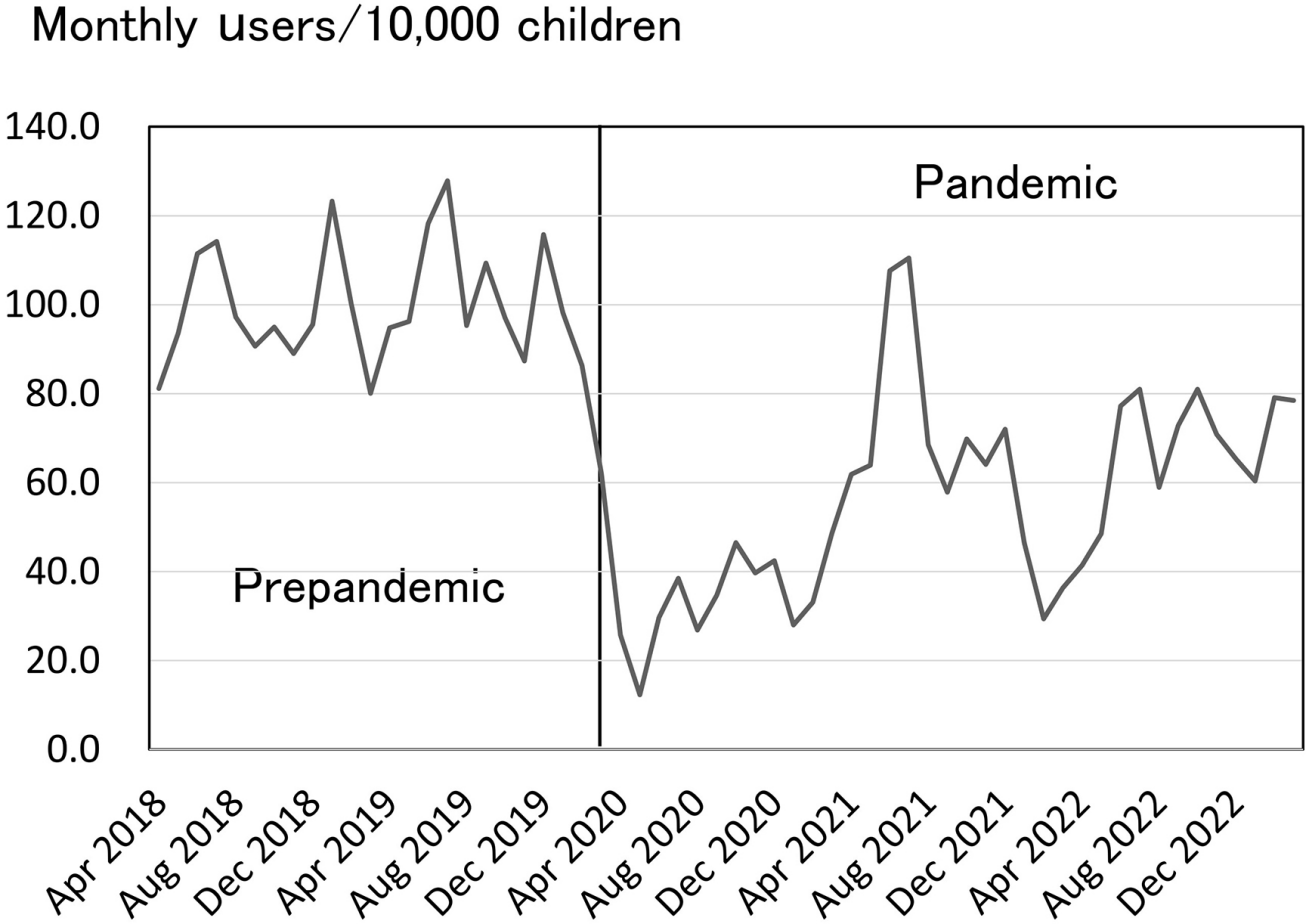
Monthly average number of users of day care services for mildly ill children per 10,000 children aged 0-6 years in 100 major cities from April 2018 to March 2023. The boundary between pre–COVID-19 pandemic and pandemic periods was defined as February 28, 2020 (vertical line). COVID-19: coronavirus disease 2019.

Responses were received from all 105 municipalities. However, municipalities lacking monthly data or those providing fewer than 10 months of data were excluded. The present analysis included only data covering 5745 city-months, obtained from 100 municipalities. This data volume accounts for 91.1% of the 6300 (105 × 60) city-month data points surveyed. Figure 2 shows the monthly average number of users of day care services for mildly ill children per 10,000 children aged 0-6 years, revealing a significant decline in user numbers since March 2020. However, calculating the monthly average number of reported cases at sentinel sites before and during the COVID-19 pandemic revealed that the number of cases of the aforementioned 13 common infectious diseases also decreased during the pandemic period (Table 2).

**Table 2.** Trends in the Occurrence of Common Infectious Diseases (Average Number of Cases/Sentinel Site/Month).

Diseases	Before the pandemic	During the pandemic
Influenza (excluding avian influenza and novel influenza or re-emerging influenza; x_4it_^*^)	21.76	3.80
Respiratory syncytial virus infection (x_5it_)	3.54	3.12
Pharyngoconjunctival fever (x_6it_)	1.86	0.66
Group A streptococcal pharyngitis (x_7it_)	9.68	2.22
Infectious gastroenteritis (x_8it_)	24.30	15.81
Varicella (x_9it_)	1.62	0.45
Hand, foot, and mouth disease (x_10it_)	7.13	2.29
Erythema infectiosum (x_11it_)	2.37	0.10
Exanthema subitum (x_12it_)	1.91	1.61
Herpangina (x_13it_)	2.61	0.85
Mumps (x_14it_)	0.47	0.18
Epidemic keratoconjunctivitis (x_15it_)	2.87	0.84
Acute hemorrhagic conjunctivitis (x_16it_)	0.04	0.02

*The notations from “x_4it_” to “x_16it_” are based on the description in the Materials and Methods section.

Table 3 presents the results of panel data analysis regarding the monthly number of users of day care services for mildly ill children. A negative correlation was observed between the number of users and the dummy variables indicating the COVID-19 pandemic or a declaration of state emergency/priority infection control measures, demonstrating a decrease in the number of users during these periods. Furthermore, there was a positive correlation between the logarithmic values of childcare capacity and the number of users.

**Table 3.** Panel Data Analysis Explaining the Monthly Number of Users of Day Care Services for Mildly Ill Children.

Variables	Coefficient	IRR	95% CI
COVID-19 pandemic dummy variable (x_0t_; prepandemic = 0, pandemic = 1)	a_0_	0.506*	0.399-0.641
Dummy variable for declaration of state emergency/priority infection control measures (x_1it_; none = 0, present = 1)	a_1_	0.801*	0.775-0.828
Average minimum distance from residence to nurseries for mildly ill children (x_2it_)	a_2_	0.825*	0.693-0.982
Interaction between pandemic dummy variable and average minimum distance (x_0t_ × x_2it_)	b_2_	1.017	0.938-1.103
Natural logarithm of nursery capacity (x_3it_)	a_3_	1.808*	1.772-1.845
Influenza (excluding avian influenza and novel influenza or re-emerging influenza; x_4it_^#^)	a_4_	1.002*	1.001-1.002
Respiratory syncytial virus infection (x_5it_)	a_5_	1.013*	1.009-1.017
Pharyngoconjunctival fever (x_6it_)	a_6_	1.019*	1.010-1.028
Group A streptococcal pharyngitis (x_7it_)	a_7_	1.004	0.999-1.009
Infectious gastroenteritis (x_8it_)	a_8_	1.004*	1.002-1.007
Varicella (x_9it_)	a_9_	1.000	0.983-1.018
Hand, foot, and mouth disease (x_10it_)	a_10_	1.004*	1.003-1.005
Erythema infectiosum (x_11it_)	a_11_	1.001	0.995-1.007
Exanthema subitum (x_12it_)	a_12_	1.032	0.994-1.071
Herpangina (x_13it_)	a_13_	1.011*	1.008-1.015
Mumps (x_14it_)	a_14_	1.020	0.986-1.055
Epidemic keratoconjunctivitis (x_15it_)	a_15_	1.008*	1.002-1.015
Acute hemorrhagic conjunctivitis (x_16it_)	a_16_	1.031	0.955-1.112
Products of the COVID-19 pandemic dummy variable and incidence rates			
Influenza (excluding avian influenza and novel influenza or re-emerging influenza, x_0t_ × x_4it_)	b_4_	1.003*	1.001-1.004
Respiratory syncytial virus infection (x_0t_ × x_5it_)	b_5_	1.020*	1.015-1.025
Pharyngoconjunctival fever (x_0t_ × x_6it_)	b_6_	1.058*	1.030-1.086
Group A streptococcal pharyngitis (x_0t_ × x_7it_)	b_7_	1.005	0.996-1.014
Infectious gastroenteritis (x_0t_ × x_8it_)	b_8_	1.008*	1.005-1.012
Varicella (x_0t_ × x_9it_)	b_9_	1.000	0.953-1.050
Hand, foot, and mouth disease (x_0t_ × x_10it_)	b_10_	1.008*	1.004-1.011
Erythema infectiosum (x_0t_ × x_11it_)	b_11_	1.075*	1.013-1.142
Exanthema subitum (x_0t_ × x_12it_)	b_12_	0.928*	0.889-0.969
Herpangina (x_0t_ × x_13it_)	b_13_	1.024*	1.012-1.035
Mumps (x_0t_ × x_14it_)	b_14_	1.194*	1.069-1.334
Epidemic keratoconjunctivitis (x_0t_ × x_15it_)	b_15_	1.009	0.982-1.038
Acute hemorrhagic conjunctivitis (x_0t_ × x_16it_)	b_16_	1.040	0.890-1.215
Natural logarithm of population aged 0 to 6 years (x_17i_)	a_17_	1	NA
Constant	c_0_	0.001	0.000-0.003

*p < 0.05. ^#^The notations from “x_4it_” to “x_16it_” are based on the description in the Materials and Methods section. CI: confidence interval; COVID-19: coronavirus disease 2019; IRR: incidence rate ratio for utilization of nurseries for mildly ill children; NA: not applicable.

Among the 13 infectious diseases, a positive correlation was observed between the number of users and the incidence rates for seven diseases; no disease showed a negative correlation. Furthermore, eight of 13 products of the COVID-19 pandemic dummy variable and infection incidence rates showed a positive correlation with the number of users. This indicates that the increase in the number of users relative to the incidence rates of these infectious diseases during the pandemic period was significantly higher for many diseases in comparison with the prepandemic period.

Analysis of the relationship between the number of users and the weighted average of the shortest distances between the place of residence and nurseries for mildly ill children revealed that for every 1-km increase in distance, the utilization rates decreased by a factor of 0.825 (dropping to approximately one-tenth at 12 km: 0.825^12^ = 0.1). However, the product of the COVID-19 pandemic dummy variable and the weighted average of the shortest distance did not show a significant correlation with the number of users. This indicates that the rate of decline in user numbers based on distance did not change significantly between the prepandemic and pandemic periods.

## Discussion

The results of this study were largely consistent with preliminary analyses conducted in a small number of large cities ^(6), (12)^, where there was a positive correlation between the incidence rates of common infectious diseases and the use of nurseries for mildly ill children. A positive correlation was also found between the number of users of nurseries and their childcare capacity. Furthermore, during the COVID-19 pandemic and when a declaration of state emergency/priority infection control measures was issued, the number of users similarly decreased. In large cities or densely populated areas, only slight differences in the modes of transportation used are expected ^(5)^. Therefore, this study focused on large cities in analyzing the relationship between utilization patterns for nurseries caring for mildly ill children and the distance to the corresponding facility. The study findings demonstrated that the utilization rates decreased by a factor of 0.825 (approximately 17.5% decrease) for every 1-km increase in distance. Defining the service area as the distance range where utilization rates become one-tenth to equivalent in comparison with childcare facilities in the same neighborhood as a child’s home, the radius remained approximately 12 km both before and during the COVID-19 pandemic. The distance of 12 km corresponds to a 30-minute journey by taxi or car, which suggests that most users consider anything within this to be a reasonable travel distance to use this childcare service.

There was no meaningful change in the range of the service area between the prepandemic and pandemic periods. One possible explanation for this is that parents who were unable to work remotely during the pandemic may have had to travel the same distance as before to access these childcare services.

A survey of all local governments nationwide that provide financial assistance for day care provided to mildly ill children found no significant correlation between the shortest distance from the residence to a nursery for mildly ill children and the number of users ^(13)^. This is thought to be because transportation to such facilities differs between urban and rural areas ^(5)^. Therefore, the present study was limited to large municipalities.

When childcare services become unavailable owing to infectious diseases, support for children and their guardians is disrupted. Ensuring continuity of childcare is a critical challenge, regardless of whether children are healthy or have mild symptoms of illness. To achieve this, it is essential to quantitatively determine the utilization patterns of day care services for mildly ill children.

This study has the following limitations. I only analyzed the relationship between the weighted average of the shortest distances from the residence to the nearest childcare facilities for sick children and the use of such facilities. However, the road distance traveled when moving 1 km in a straight line across all 112 major cities in Japan with populations exceeding 200,000 was 1.3035 km, on average (minimum 1.1260 km, maximum 1.9235 km), and the coefficient of determination between the road distance and straight-line distance ranged from 0.8937 to 0.9851 ^(14)^. Therefore, the straight-line distance appears to reflect the road distance traveled.

The analysis showed that the product of the COVID-19 pandemic dummy variable and the weighted average of the shortest distances did not show a significant correlation with the number of users. However, this null finding may also be attributable to limited statistical power to detect modest changes in the distance–utilization relationship.

Analysis treating the period from January 2020 (when Japan’s first COVID-19 case was confirmed) as the pandemic period showed a 17.5% decrease (0.825 times) in facility users per kilometer. Furthermore, when the pandemic period was defined as starting after the first state of emergency was declared in April 2020, facility user numbers decreased by 14.3% (0.857 times). The reduction rates for both differed significantly from 0% (p < 0.05). However, this represents only a slight difference in comparison with the initial analysis, in which the period from March 2020 onward was defined as the pandemic period where the rate of decrease in users per kilometer was 17.5% (0.825 times). A significant negative correlation between the number of users and the product of the COVID-19 pandemic dummy variable and weighted average of the shortest distance was observed only when the period from April 2020 onward was defined as the pandemic period. Independently of the relationship between distance and the number of users, the utilization rates decreased further by 6.2% (0.938 times) per kilometer during the pandemic period. It is likely that during the COVID-19 pandemic, these childcare facilities were rarely fully occupied, making it possible to use facilities close to home. However, because a sharp decline in user numbers occurred after March 2020, the pandemic was considered to have begun in March 2020 in the present analysis.

The study sample includes three types of major cities: 20 designated cities, 23 special wards of Tokyo, and 62 core cities. These city types may differ substantially in terms of population density, public transportation availability, and childcare infrastructure. However, further stratification according to environmental factors, so as to reduce the number of municipalities, would make it difficult to detect significant differences; therefore, the analysis sample was not reduced further.

The changes in parents’ thresholds for placing mildly ill children in childcare facilities during the pandemic, as well as shifts in the composition of children attending regular childcare facilities, remain unclear. However, during the COVID-19 pandemic, a high proportion of users’ parents were health care professionals (doctors and nurses) ^(15)^. Therefore, it cannot be ruled out that the use threshold may differ between the prepandemic period and the pandemic period.

It seems likely that when the nearest facility is full, parents might seek childcare elsewhere. Although the distance from the second-closest facility to the nearest one was not analyzed in this study, in approximately half of childcare facilities for mildly ill children, another facility is located within a 3-km radius ^(16)^.

### Conclusions

Defining the service area for nurseries serving mildly ill children in 105 major Japanese cities as the distance range where utilization rates become one-tenth to equivalent, compared with childcare facilities in the same neighborhood as a child’s home, the radius remained approximately 12 km both before and during the COVID-19 pandemic.

## Article Information

### Acknowledgments

I received valuable advice from Professor Yoshinori Kawasaki, Institute of Statistical Mathematics, and I would like to thank Analisa Avila, MPH, ELS, of Edanz (https://jp.edanz.com/ac) for English language editing.

### Author Contributions

Akira Ehara was responsible for the conception and design of this study, data collection, analysis, interpretation, drafting of the manuscript, and approval of the final version to be published.

### Conflicts of Interest

The authors declare that there are no conflicts of interest. Akira Ehara is not a member of the Editorial Board of this journal.

### Institutional Review Board Approval Number and Name of the Institution

23-012, Hiroshima International University

### Participant Consent

This research did not involve human subjects and therefore did not require consent from participants or their guardians.
